# Correlation between pathologic complete response, event-free survival/disease-free survival and overall survival in neoadjuvant and/or adjuvant HR+/HER2-breast cancer

**DOI:** 10.3389/fonc.2023.1119102

**Published:** 2023-05-02

**Authors:** Anagha Gogate, Sandip Ranjan, Amit Kumar, Hitesh Bhandari, Eros Papademetriou, Inkyu Kim, Ravi Potluri

**Affiliations:** ^1^ WWHEOR, Bristol Myers Squibb, Princeton, NJ, United States; ^2^ HEOR, SmartAnalyst India Pvt. Ltd., Gurgaon, India; ^3^ HEOR, SmartAnalyst Inc., New York, NY, United States

**Keywords:** HR+/HER2- breast cancer, neoadjuvant/adjuvant, overall survival, surrogate endpoints, surrogate threshold effect

## Abstract

**Purpose:**

The study’s purpose was to evaluate the correlation between overall survival (OS) and its potential surrogate endpoints: pathologic complete response (pCR) and event-free survival (EFS)/disease-free survival (DFS) in neoadjuvant and/or adjuvant HR+/HER2- breast cancer.

**Methods:**

Systematic search was performed in MEDLINE, EMBASE, Cochrane Library databases and other relevant sources to identify literature that have reported outcomes of interest in the target setting. The strength of correlation of EFS/DFS with OS, pCR with OS, and pCR with EFS/DFS was measured using Pearson’s correlation coefficient (r) based on weighted regression analysis. For Surrogate Endpoint-True Endpoint pairs where correlation was found to be moderate, surrogate threshold effect (STE) was estimated using a mixed-effects model. Sensitivity analyses were conducted on the scale and weights used and removing outlier data.

**Results:**

Moderate correlation was observed of relative measures [log(HR)] of EFS/DFS and OS (r = 0.91; 95% CI: 0.83, 0.96, *p* < 0.0001). STE for HR_EFS/DFS_ was estimated to be 0.73. Association between EFS/DFS at 1, 2 and 3 years with OS at 4- and 5-year landmarks was moderate. Relative treatment effects of pCR and EFS/DFS were not strongly associated (r: 0.24; 95% CI: -0.63, 0.84, *p* = 0.6028). Correlation between pCR and OS was either not evaluated due to inadequate sample size (relative outcomes) or weak (absolute outcomes). Results obtained in the sensitivity analyses were similar to base scenario.

**Conclusion:**

EFS/DFS were moderately correlated with OS in this trial-level analysis. They may be considered as valid surrogates for OS in HR+/HER2- breast cancer.

## 1 Introduction

Breast cancer, arising in the epithelial cells of ducts and lobules of the mammary glands, is the most prevalent cancer in women worldwide, with 7.8 million cases diagnosed in the last five years, and 685,000 breast cancer related deaths reported globally in 2020 (1). Within the various molecular subtypes of breast cancer (2), hormone receptor-positive (HR+)/human epidermal growth factor receptor 2-negative (HER2-) breast cancer is predominant, accounting for 65% of cases in females <50 years of age, and 75% of cases among females >50 years (3, 4). Both adjuvant (surgery followed by irradiation, systemic treatment, and hormonal therapy) and neoadjuvant (pre-operative treatment of tumors with systemic therapy) treatment regimens are effective in improving outcomes in HR+/HER2- patient population (5).

Neoadjuvant treatment has established itself over the past three decades as an equally effective option as adjuvant treatment, but with the added benefit of increased rates of breast-conserving surgery (6). Several studies have highlighted the significant role of neoadjuvant endocrine therapy in HR+/HER2− breast cancers, especially in the postmenopausal setting (7–9). The treatment landscape for HR+/HER2- early breast cancer patients has developed further in recent years, with the introduction and rapid adoption of targeted agents (e.g. CDK4/6 inhibitors such as abemaciclib and PARP inhibitors such as olaparib).

Development of these noveltherapies in recent times has increased the number of clinical trials focused on HR+/HER2- breast cancer. Clinical endpoints evaluating specific outcomes are fundamental to clinical trials, for assessing the efficacy, safety, and generalizability of cancer-targeted therapy (10, 11). Overall survival (OS, defined as the time from randomization until death from any cause) has long been the ‘gold standard endpoint’ in clinical trials in oncology, since it directly estimates the effect of an intervention on patient’s survival, the ultimate goal of cancer therapies (10). Simplicity of evaluation and estimation free from researchers’ bias are factors that have helped establish OS as a primary endpoint in oncology trials (12). However, OS estimation can be challenging, particularly in malignancies that have long survival, such as breast cancers. Determination of OS often mandates lengthy follow-ups, and deaths unrelated to cancer can confound the OS rates (13). Furthermore, variables such as therapy crossover, and post-progression therapies can also influence treatment effect on OS over time. These factors make it difficult to identify the underlying treatment effect (13, 14).

As OS is the most important benefit of the intervention to the patient, it is regarded as the ‘true’ endpoint (TE). However, given the challenges in measuring OS, surrogate endpoints (SEs) that are objectively measured and evaluated and predict clinical benefit (or harm or lack of benefit or harm) based on epidemiologic, therapeutic, pathophysiologic, or other scientific evidence (15) are often employed in clinical trials. SEs shorten the time required for therapeutic evaluation, involve lower costs, and require a smaller sample size compared to true endpoints (16, 17), and are therefore more practical in assessment of treatment efficacy (18, 19). Correlation between SEs and TE is however essential to correctly assess the effect of a treatment on the true endpoint, and to validate the applicability of an SE as the focal endpoint in a clinical trial. Several approaches have been proposed for surrogate validation (20). The method described by Prentice based on four major criteria is the most commonly used to validate SEs (21), the others being guidelines from German Institute for Quality and Efficiency in Health Care (IQWiG) (22), and the biomarker surrogate evaluation schema (BSES) (23).

In the context of HR+/HER2- breast cancers, potential SEs include disease-free survival (DFS), event-free survival (EFS), and pathologic complete response (pCR). These endpoints, along with others such as objective response rate, progression-free survival and time to progression, have been now recognized by the United States Food and Drug Administration (US FDA) as standard clinical endpoints in trials on novel cancer interventions seeking accelerated regulatory approval (24). pCR is the earliest available endpoint in clinical studies; however, the use of pCR as a reliable surrogate is not yet established in HR+ disease. In a recent study on HER2- breast cancer patients, the association of pCR with improved OS has been reported, however this correlation has not been analyzed statistically (25). Gyawali et al. found a moderate correlation between EFS and OS in their study on early breast cancers under neoadjuvant settings (19). Similar association of SE with OS have been explored in other studies involving breast cancer in the adjuvant and neoadjuvant settings involving molecular subtypes other than HR+/HER2-, and colorectal cancer (13, 26, 27). However, there is a paucity of correlation studies between SEs and OS specifically in the adjuvant/neoadjuvant settings in HR+/HER2- breast cancer patient population.

To address this gap, this study was conducted with the aim of extracting trial-level outcomes data from a systematic review of literature reporting these endpoints, followed by correlation analysis of pCR with OS and of EFS/DFS with OS in HR+/HER2- breast cancer patient population. The study also evaluates correlation of pCR with EFS/DFS, as pCR requires a relatively shorter evaluation period. Findings of this study will aid in strengthening the evidence base on clinical endpoints in HR+/HER2- breast cancer trials and support the accelerated evaluation of novel interventions for better disease management.

## 2 Materials and methods

### 2.1 Literature search strategy

To identify publications that have reported on the target endpoints in the patient population of interest, a systematic review of published literature was carried out in accordance with Preferred Reporting Items for Systematic reviews and Meta-Analyses (PRISMA) guidelines (28). MEDLINE (via OvidSP), EMBASE (via OvidSP), and the Cochrane Library (via Cochrane) databases were electronically searched for the time period from January 1, 2000 through January 5, 2021, using population intervention, comparison, outcomes, and study (PICOS)-based predefined search strategy (**Supplementary Table S1**). Relevant citations were retrieved using MeSH terms/keywords related to HR+/HER2- breast cancer, adjuvant, neoadjuvant, pCR, DFS, EFS, OS, and their aliases. Detailed search strategies employed for the research have been provided in **Supplementary Table S2**. Other sources such as conference proceedings (last three years) from American Society of Clinical Oncology (ASCO), European Society of Medical Oncology (ESMO), Miami Breast Cancer Congress, San Antonio Breast Cancer Symposium and European Breast Cancer Congress; clinical trial registries including Clinicaltrials.gov, EU Clinical Trials Register, ISRCTN registry; and bibliographies from relevant systematic reviews were manually searched for relevant publications.

### 2.2 Study selection

Studies were selected for inclusion if they met each of the following pre-defined criteria: (i) clinical studies with a sample size >30, including randomized clinical trials, non-randomized clinical studies, single-arm trials, and/or prospective or retrospective observational studies, (ii) patients with HR+/HER2- breast cancer, (iii) any treatment in the neoadjuvant and/or adjuvant settings; (iv) studies reporting at least one of the following outcomes: pCR, EFS, DFS, and OS; (iv) studies in English language. Non-human studies, studies without relevant outcomes, case studies, case reports, case series, letters to editor, commentaries, opinion and systematic reviews and meta-analysis, and non-English publications were excluded. Details of these selection criteria have been provided in **Supplementary Table S1**.

### 2.3 Data extraction for assessment

Dual review system involving two independent researchers was employed at all stages of study selection and data collection. Preliminary screening was carried out on titles and abstracts, followed by screening of full texts before data extraction. Outcomes data in the form of hazard ratios and survival estimates at landmark time-points (one year (1-yr), two years (2-yr), three years (3-yr), four years (4-yr) and five years (5-yr)) for time-to-event outcomes (OS, DFS, EFS), odds ratios and proportions for pCR were collated from the identified studies.

For studies that involved a comparative trial but did not report hazard ratios (HRs) of outcomes of the intervention with respect to a comparator, these were derived with the help of Cox regression models using pseudo-individual patient data (IPD) created by applying Guyot’s algorithm to the digitized Kaplan–Meier curves (29). For studies not reporting pCR rates or survival rates at landmark time points for the overall HR+/HER2- population, the outcomes were estimated as weighted averages of estimates for relevant subgroups. DFS and EFS were analyzed as a single outcome measure as these definitions were seen to be used interchangeably in the studies identified in SLR – their definition has been provided in **Supplementary Table S3**.

### 2.4 Statistical analysis

Weighted regression approach was used for quantitative synthesis of evidence from included studies. Correlation analyses were conducted for the following SE-TE pairs: (i) EFS/DFS and OS, (ii) pCR and EFS/DFS (iii) pCR and OS - the analyses were carried out for both relative and absolute outcomes.

The correlation analysis of relative treatment effect between SE and TE was carried out in terms of logarithmic transformation of HRs for time-to-event outcomes (DFS, EFS and OS), and logarithmic transformation of odds ratios (ORs) for the response outcome (pCR), as similarly done in a previous study (30). Correlation of absolute treatment effects was calculated in terms of survival probability at landmark time points for the time-to-event outcomes and as proportion for pCR.

For the weighted regression analysis, weights were based on sample size. The strength of linear association between each SE-TE pair was evaluated in terms of Pearson’s correlation coefficient (r), while coefficient of determination (R^2^) was used for quantification of heterogeneity accounted for by the regression model. In addition, White Test was used to determine heteroscedasticity, with values > 0.05 indicating no heteroscedasticity of data. All statistical analyses were performed using SAS 9.4 (SAS Institute Inc.) and R (version 4.1.0) software.

IQWiG recommends that validity of a candidate as a SE be evaluated based on whether the correlation with the true endpoint is high (lower confidence limit (LCL) of r > 0.85) or low (upper confidence limit (UCL) of r < 0.70), respectively. In all other cases, where correlation is moderate making the validity of the SE ambiguous, the concept of surrogate threshold effect (STE) is applied to draw conclusions on the surrogacy of the SE (31). STE represents the minimum value of treatment effect (i.e., maximum value of HR in our context) for SE that needs to be observed in a trial to permit drawing a conclusion of a non-zero significant effect on the true endpoint (32). In this study, STE was estimated by applying a mixed-effects model to the data with HR of EFS/DFS as moderator and HR of OS as an outcome variable. Standard error of EFS/DFS was used as weight for this analysis. Heterogeneity was estimated using the restricted maximum likelihood (REML) estimator for heterogeneity (33).

### 2.5 Sensitivity analysis

Several sensitivity analyses were conducted to evaluate the impact of potentially influential parameters on overall findings: (i) alternate transformations were analyzed by applying linear transformations instead of logarithmic transformations for relative outcomes, (ii) for relative outcomes, trial sample size was replaced by inverse of variance as weights, (iii) analyses were also carried out by excluding studies that did not define pCR as absence of invasive cancer in breast and axillary nodes, irrespective of ductal carcinoma in-situ (ypT0/is ypN0), (iv) correlation analyses were carried out by removing outlier data, identified by visual inspection, (v) in case of STE estimations using mixed-effects model, standard error of OS was used as the weight rather than the standard error of the moderator variable for sensitivity analysis.

## 3 Results

### 3.1 Selection of studies

**Figure 1:**
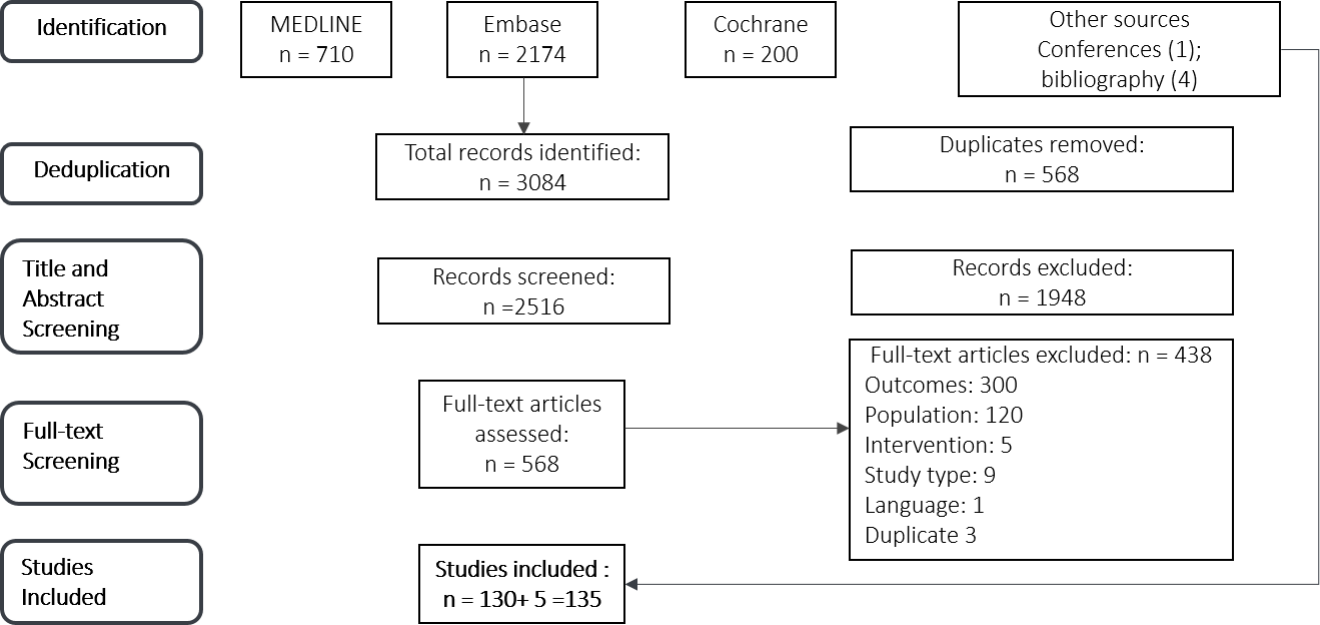
PRISMA flow-chart of study selection n, number of studies.

A total of 3,084 studies were identified from database search (MEDLINE n = 710; Embase, n = 2,174; Cochrane, n = 200), of which 2,516 studies were excluded based on duplications and review of title and abstract (**Figure 1**). After the full-text screening of the remaining 568 studies, 130 were deemed eligible for inclusion in this study, while 438 studies were excluded for various reasons captured in **Figure 1**. In addition, five studies from conference abstracts and bibliographies of relevant citations were identified as eligible and included in the final list of studies for analysis.

### 3.2 Correlation between EFS or DFS and OS

**Figure 2:**
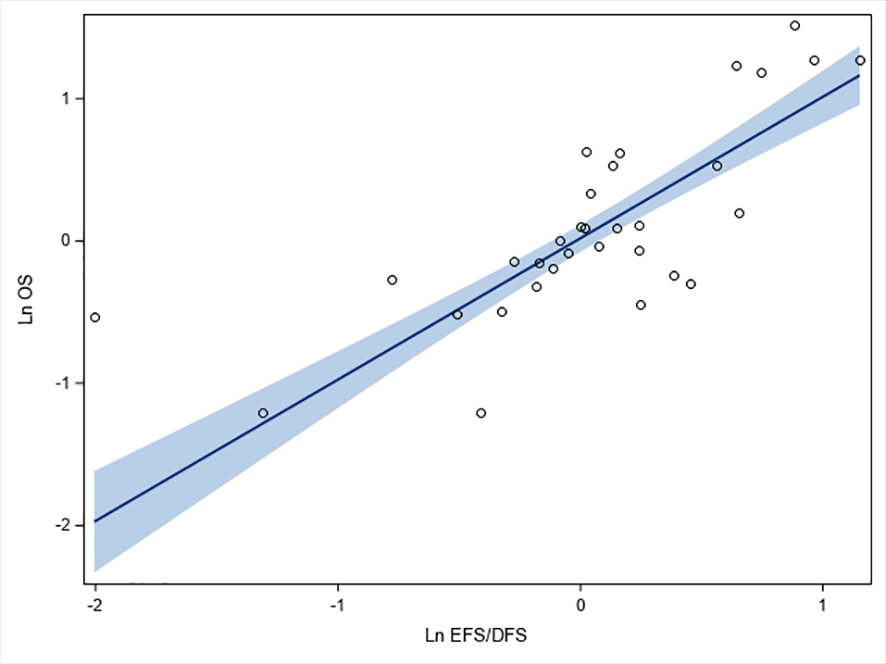
Fit plots and fit statistics for correlation between log HR for OS and log HR for EFS/DFS. Each individual circle of the graph represents HR data point from a single study. Solid blue line indicates the fitted weighted linear regression line. DFS, disease-free survival; EFS, event-free survival; Ln, natural logarithm; OS, overall survival.

**Figure 2** shows the correlation between the HR of EFS/DFS and HR of OS on logarithmic scale, reported in 32 observations involving 35,543 HR+/HER2- breast cancer patients. Moderate correlation was observed between EFS/DFS and OS (r: 0.91, 95% CI: 0.83, 0.96, *p* < 0.0001; R^2^: 0.83; 95% CI: 0.69, 0.89). The linear relationship between endpoints was positive, with a one-unit increase in log (HR) of EFS/DFS corresponding to a 0.99-unit increase in log (HR) for OS (beta coefficient: 0.99; 95% CI: 0.83, 1.16; *p* < 0.0001) (**Figure 2**). Heteroscedasticity of the studies evaluated by White Test revealed no heteroscedasticity (*p* = 0.79), indicating the absence of outliers in the analyzed data.

**Figure 3:**
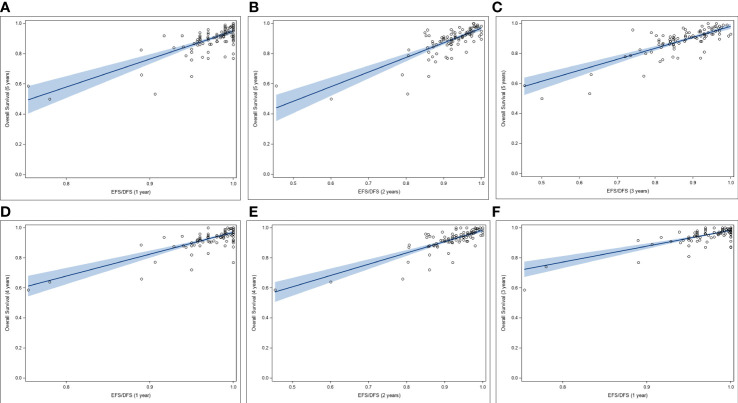
Correlation between rates of OS and rates of EFS/DFS at landmark time points **(A-C)**, Correlation between 5-yr OS rates and **(A)** 1-yr, **(B)** 2-yr, and **(C)** 3-yr rates of EFS/DFS. **(D, E)**, Correlation between 4-yr OS rates and **(D)** 1-yr, and **(E)** 2-yr rates of EFS/DFS. **(F)** Correlation between 3-yr OS rates and 1-yr rates of EFS/DFS. DFS, disease-free survival; EFS, event-free survival; OS, overall survival.

In this systematic review, to assess EFS/DFS as potential SE for 5-yr and 4-yr OS in HR+/HER2- breast cancer, correlation analyses were performed between rates of EFS/DFS and OS at landmark time-points using data aggregated from 92 studies. A moderate correlation was observed between each of 1-yr, 2-yr, and 3-yr EFS/DFS rates, and 5-yr OS, with r of 0.69, 0.75, and 0.76, respectively. Moreover, the correlations were statistically significant (*p* < 0.0001) in all cases. No heteroscedasticity was observed for any of the above-mentioned correlations (White Test *p*-values ranging from 0.21 to 0.29) (**Figures 3A–C**). EFS/DFS rates at 1-yr and 2-yr landmark points correlated moderately with 4-yr OS rates (r of 0.69 and 0.71, respectively). The associations were statistically significant (*p* < 0.0001) (**Figures 3D, E**). Similarly, moderate association was observed between 1-yr EFS/DFS rates with 3-yr OS rates (**Figure 3F**). In all cases, positive linear associations were found between the absolute outcomes for the landmark time points, and no heteroscedasticity was noted for any of the analyzed data sets.

### 3.3 Correlation between pCR and OS

Correlation analysis could not be carried out between OR of pCR and HR of OS as the number of observations was low (n=3). For absolute treatment outcomes, no correlation was observed between pCR and OS at any of the landmark time points (1-yr, 2-yr, 3-yr, 4-yr and 5-yr OS) from regression analysis of data of 31-34 included studies involving 19,385 to 23,192 HR+/HER2- breast cancer patients (**Supplementary Table S4**, **Supplementary Figure S1**). R^2^ for this association ranged between 0.02 to 0.11, and r from 0.15 to 0.33. Although the linear association between the pCR and OS was positive, it was not statistically significant for any of the landmark time points (**Supplementary Table S4**).

### 3.4 Correlation between pCR and EFS or DFS

**Figure 4:**
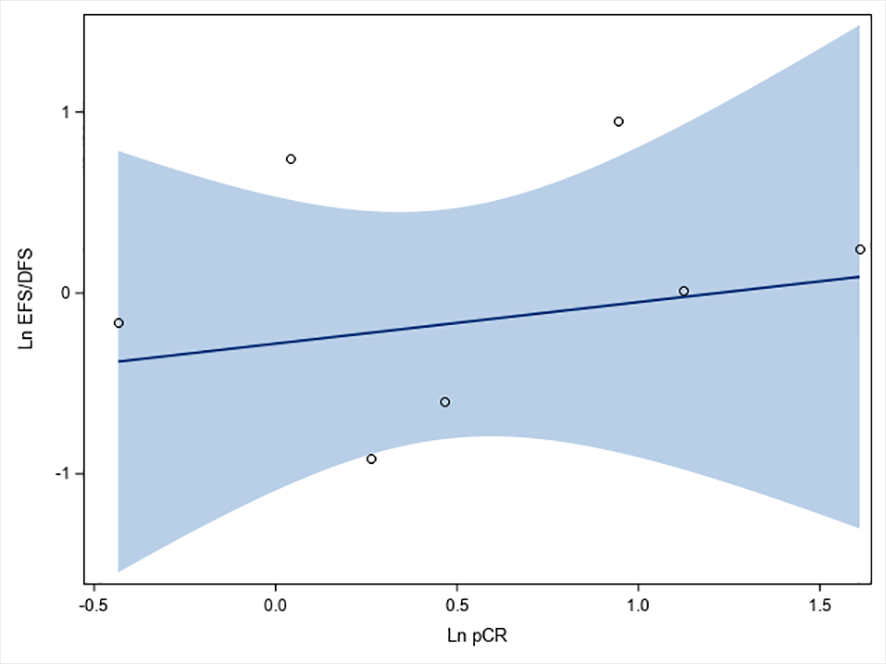
Fit plots and fit statistics for correlation between log OR for pCR and log HR for EFS/DFS. Each individual circle of the graph represents HR data point from a single study. Solid blue line indicates the fitted weighted linear regression line. DFS, disease-free survival; EFS, event-free survival; Ln, natural logarithm; OS, overall survival.

Regression analysis of data obtained from seven studies involving 1,571 HR+/HER2- breast cancer patients showed that log (OR) of pCR correlated minimally with log (HR) of EFS/DFS, and the association was not statistically significant (r: 0.24, 95% CI: -0.63, 0.84, *p* = 0.6028; R^2^: 0.056, 95% CI: 0.00, 0.47) (**Figure 4**). Positive linear association was observed between log(OR) of pCR and log(HR) of EFS/DFS (beta coefficient: 0.23, 95% CI: -0.86, 1.31, *p* = 0.6099), although the lower bound of the confidence interval was negative. No heteroscedasticity was observed (*p* = 0.1688, which is > 0.05) (**Figure 4**).

There was no correlation between survival estimates at 2-yr and 5-yr landmark time points for EFS/DFS and pCR (**Supplementary Figures S2A, B**), and the correlations were not statistically significant (**Supplementary Table S5**).

### 3.5 Surrogate threshold effect analysis

**Figure 5:**
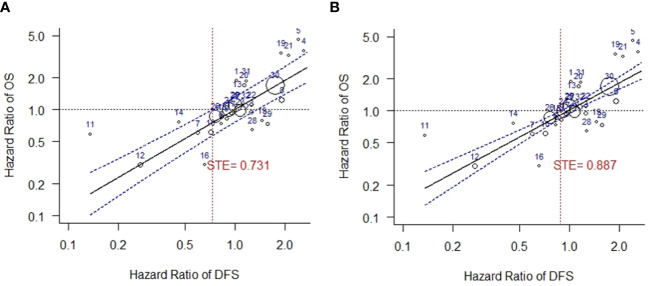
Surrogate threshold effect (STE) analysis for DFS vs. OS. **(A)** STE analysis with variance of HR of DFS as weights. **(B)** STE analysis with variance of HR of OS as weights. DFS, disease-free survival; OS, overall survival; STE, surrogate threshold effect.

In this study, the correlation between log HR of EFS/DFS and outcome variable log HR of OS was moderate (lower limit of CI for r = 0.83), and therefore to establish the validity of EFS/DFS as a surrogate for OS, STE analysis was conducted. STE for HR of DFS was observed to be 0.731, when HR of EFS/DFS and HR of OS were weighted by moderator outcome (DFS) variance. (**Figure 5A**). The results were statistically significant (*p* < 0.001 for beta DFS) and low residual heterogeneity was observed using the REML estimator for heterogeneity (τ^2 ^= 0.0178, I^2 ^= 36.00%).

A higher STE was obtained when analysis was carried out using variance of OS as weights in the mixed-effects model. Heterogeneity was not detected in this analysis (STE = 0.887, τ^2 ^= 0, I^2 ^= 0.00%, *p* < 0.001 for beta DFS) (**Figure 5B**).

### 3.6 Sensitivity analysis

Results obtained in the sensitivity analyses for correlations between SEs and OS were consistent with the results obtained for the base scenario. The association between OR of pCR and HR of EFS/DFS remained weak despite changed scenarios and removal of outliers (**Table 1A**, **Supplementary Table S6**). Similar results were obtained for landmark time points of pCR versus OS (**Table 1B**). Weak association, similar to the base case, was observed between pCR rates at landmark time points and EFS/DFS after sensitivity analysis (**Supplementary Table S7**).

**Table 1A:** Results of sensitivity analysis.

Scenario	Dataset	Scale	Weights	R^2^ (95% CI)	r (95% CI)	*p*-value
**Between HR of OS and HR of EFS/DFS**						
Base Case	Full dataset	Log	Sample size	0.83 (0.69, 0.89)	0.91 (0.83, 0.96)	<.0001
Sensitivity 1	Full dataset	Log	Inverse of variance	0.76 (0.57, 0.83)	0.87 (0.75, 0.93)	<.0001
Sensitivity 2	Full dataset	Linear	Sample size	0.82 (0.68, 0.88)	0.91 (0.82, 0.95)	<.0001
Sensitivity 3	Without outliers	Log	Sample size	0.86 (0.73, 0.91)	0.93 (0.85, 0.96)	<.0001
**Between OR of pCR and HR of EFS/DFS**						
Base Case	Full dataset	Log	Sample size	0.06 (0, 0.47)	0.24 (-0.63, 0.84)	0.61
Sensitivity 1	Full dataset	Log	Inverse of variance	0.07 (0, 0.48)	0.26 (-0.61, 0.85)	0.58
Sensitivity 2	Full dataset	Linear	Sample size	0.06 (0, 0.47)	0.24 (-0.62, 0.84)	0.60
Sensitivity 3	Without outliers	Log	Sample size	0.02 (0, 0.41)	0.12 (-0.76, 0.85)	0.81

CI, confidence interval; DFS, disease-free survival; EFS, event-free survival; HR, hazard ratio; OS, overall survival; R^2^, coefficient of determination; r, Pearson’s correlation coefficient.

**Table 1B:** Sensitivity analyses for OS at landmark time points vs. pCR.

Model	Scenario	R^2^ (95% CI)	r (95% CI)	*p*-value
pCR vs 3-yr OS	Full dataset	0.02 (0, 0.18)	0.14 (-0.21, 0.46)	0.43
	pCR = ypT0/is ypN0	0.01 (0, 0.18)	0.09 (-0.31, 0.46)	0.66
	After removing outliers	0.03 (0, 0.22)	0.18 (-0.19, 0.51)	0.34
pCR vs 4-yr OS	Full dataset	0.03 (0, 0.22)	0.18 (-0.17, 0.5)	0.3031
	pCR = ypT0/is ypN0	0.01 (0, 0.2)	0.12 (-0.29, 0.49)	0.5671
	After removing outliers	0.05 (0, 0.26)	0.23 (-0.15, 0.55)	0.2253
pCR vs 5-yr OS	Full dataset	0.11 (0, 0.32)	0.33 (-0.01, 0.61)	0.0583
	pCR = ypT0/is ypN0	0.1 (0, 0.33)	0.31 (-0.09, 0.62)	0.1249
	After removing outliers	0.17 (0, 0.4)	0.41 (0.05, 0.68)	0.0267

CI, confidence interval; OS, overall survival; pCR, pathological complete response; R^2^, coefficient of determination; r, Pearson’s correlation coefficient; yr, year.

## 4 Discussion

Early availability of surrogate endpoints with a high level of reliability has the potential to reduce the time and financial burden associated with conducting clinical trials and accelerate drug development. Several new drugs have been approved by the FDA and the European Medicines Agency (EMA) (34) based on treatment outcomes determined from trials that assessed SEs (35–37). Apart from facilitating regulatory endorsements, evidence from SEs enables decisions on insurance coverage and reimbursements (38). In this context, robust validation analyses of existing and new SEs become critical for the use of SEs in oncology.

Surrogacy validation studies are not uniformly relevant across cancer subtypes and settings. The reliability and relative time of availability of a SE compared with the definitive endpoint are limited and specific to the tumor type, setting (adjuvant/metastatic), line of therapy, types of agents (cytotoxic versus targeted drugs), and surrogate–clinical outcome combination. For example, in breast cancer, correlation studies between progression free survival (PFS) and OS have been carried out in exclusively triple-negative breast cancer patients and for this group of patients, PFS was found to be a valid surrogate for OS (39). Similarly, correlations of EFS/DFS with OS have been reported in early breast cancer patient populations as a whole (30), specific populations such as in patients with HER2+ breast cancer (40), in pancreatic cancer patients (41), and in colorectal cancer patient population (13, 42). Results from these studies suggest that the findings are specific to the cancer/subtype and therefore SE-TE relationships cannot be applied across clinical settings and patient populations. To date, surrogacy studies with DFS and EFS have not been reported specifically for early HR+/HER2- breast cancer, which accounts for a majority of all breast malignancies, and association of pCR with OS has been reported to be less obvious in this cancer subtype (43). This is the first study to describe trial-level relationships between distinct SE-TE combinations in HR+/HER2- breast cancer patients in the adjuvant/neoadjuvant settings.

Analysis of data from the published literature in this study shows that improvements in relative outcomes of EFS/DFS in HR+/HER2- breast cancers are moderately but statistically significantly associated with improvements in relative outcomes of OS. A moderate correlation was also observed between absolute outcomes of EFS/DFS and OS at landmark time points, and the results of the sensitivity analysis were consistent with base results. The R^2^ between EFS/DFS and OS was 0.83, indicating that 83% of the variation in OS can be explained by the variation in the EFS/DFS. These results are consistent with the results from a previous study on HR+/HER2- metastatic breast cancer patients, where correlation between log (HR) of OS and log(HR) of PFS was reported to be 0.72 (19). In other studies on different groups of breast cancer patients, correlation coefficients between relative treatment effects of SEs (such as partial or complete tumor response, disease progression, time to progression, and PFS) and OS ranged from 0.48 to 0.76, although analyzed trials included heterogeneous patient populations (44–46).

Additionally, STE analyses in this study support the validity of EFS/DFS as surrogates for OS in HR+/HER2- breast cancer. The STE approach provides a natural interpretation from a clinical standpoint, along with providing information on practical application of SEs. Several oncology studies have used this approach to validate SEs (47–49) and this method serves as a means to relay information between researchers and clinicians regarding validity of an SE.

pCR is one of the earliest available end-points in breast cancer trials, and is included in the FDA’s list of approved SEs for HER2+ breast cancer, based on findings of the NeoSphere (50) and TRYPHAENA (51) clinical trials. The current study set out to examine its correlation with DFS and EFS, as well as with OS, with a view to determining if SEs that can be evaluated the earliest in a trial, such as pCR, may be utilized instead of DFS/EFS or OS that are established later in the trial. Establishing surrogacy *via* such correlations would be invaluable for medications that are breakthrough discoveries and warrant rapid approval. Trial-level analysis in this study among HR+/HER2- breast cancer patients showed that relative treatment effects of pCR and EFS were associated, although not strongly, and results of sensitivity analysis were consistent with base results. However, there was no association between absolute outcomes of pCR and OS - the coefficient of determination ranged from 0.02 to 0.11, indicating that only 2% to 11% of the variability among treatment effects on OS can be predicted by the outcomes observed with pCR. Similar results were obtained in sensitivity analysis carried out using an alternate definition of pCR, where studies that did not define pCR as absence of invasive cancer in breast and axillary nodes, irrespective of ductal carcinoma in-situ (ypT0/is ypN0) were excluded. These findings are consistent with the trial-level analysis conducted in the Collaborative Trials in Neoadjuvant Breast Cancer (CTneoBC), a working group established by the FDA, where little association was found between increases in frequency of pCR and EFS (R²=0·03, 95% CI 0·00–0·25) and OS (R²=0·24, CI 0·00–0·70) for the overall breast cancer population. Interestingly, a responder analysis in the study showed major benefit in long-term outcomes from achieving pCR in patients, particularly with aggressive breast cancer subtypes (triple-negative; HER2+ and HR-, and high-grade HR+, HER2-negative) (27).

This study has some limitations. Patient characteristics of individual trials included in the various correlation analyses were not studied, nor any subgroup analyses based on any such characteristics conducted. Trial-level surrogacy, as estimated in this analysis, provides a measure of how well the treatment effect on a surrogate endpoint predicts the treatment effect on a true endpoint at a group level and is not based on and not reflective of surrogacy at individual patient data level (49). Only published data have been included, and there is a likelihood that unpublished literature or studies that have been excluded in this systematic review due to lack of data may have poorer correlations. Importantly, existing neoadjuvant studies have limited power to detect differences in EFS/DFS and OS because they were powered to measure pCR rate differences that could limit detecting any association with pCR. Furthermore, HR+/HER2- breast cancer is no longer considered to be a homogeneous disease subset. Molecular assays now can distinguish between HR+/HER2- breast cancers that benefit, or not, from adjuvant chemotherapy. In the current analysis, we could not analyze separately the surrogate function of pCR for EFS/DFS and OS in the chemotherapy sensitive subset (i.e. high recurrence score, luminal B cancers) of HR+ cancers. Studies with or without crossover have not been separately considered, and the use of crossover in clinical trials may change the association between SEs and OS. In addition, no studies involving targeted therapies or immune-oncology agents were identified in the evidence base of the current study due to their more recent entry in the field of cancer therapy. We cannot be certain if the conclusions regarding the associations between the TE and SEs derived from historical data can be extrapolated to patient populations receiving targeted adjuvant/neoadjuvant therapies or immune-oncology agents.

## Conclusion

In conclusion, EFS and DFS are appropriate surrogate endpoints in adjuvant and neoadjuvant clinical trials of HR+/HER2- breast cancer patients. Our results support using these metrics to seek regulatory approval and reimbursement. On the other hand, based on the currently available data, pCR does not serve as a reliable predictor of OS or of EFS/DFS in HR+/HER2- breast cancers; however more advanced molecular subtyping and patient selection in future studies could change this.

## Data availability statement

The original contributions presented in the study are included in the article/**Supplementary Material**. Further inquiries can be directed to the corresponding author.

## Author contributions

AG and IK: Study design, analysis review, and manuscript writing. SR, AK and HB: Study design, literature review, data extraction, data analysis, and manuscript writing. EP: Data analysis, and manuscript writing. RP: Study design, analysis review, and manuscript writing. All authors contributed to the development of the manuscript. All authors contributed to the article and approved the submitted version.
