# Supplementary material for: Correlation between pathologic complete response, event-free survival/disease-free survival and overall survival in neoadjuvant and/or adjuvant HR+/HER2-breast cancer
Source: Front Oncol. 2023 May 2;13:1119102. doi: 10.3389/fonc.2023.1119102 (PMC10185900; doi:10.3389/fonc.2023.1119102)
Supplement: Supplementary file 1 [file DataSheet_1.docx]

Supplementary Material

**Supplementary Table S1:** Population, intervention, comparison, outcomes, and study (PICOS)-based inclusion and exclusion criteria for studies included in the systematic review

**Supplementary Table S2:** Search strategy used for literature search

1. Search strategy used for Medline (via Ovid) searched on January 5, 2021
2. Search strategy used for Embase (via Ovid) searched on January 5, 2021
3. Search strategy used for Cochrane (via Cochrane Library) searched on January 5, 2021

**Supplementary Table S3:** Definitions of DFS, EFS, RFS, etc. used across various studies

**Supplementary Table S4:** Correlation between OS at yearly landmark time points and pCR

**Supplementary Table S5:** Correlation between DFS/EFS at yearly landmark time points and pCR

**Supplementary Table S6:** Sensitivity analysis of correlation between OS and DFS/EFS at landmark time points

**Supplementary Table S7:** Sensitivity analysis of correlation between pCR and DFS at landmark time points

**Supplementary Figure S1:** Correlation between rates of OS at landmark time points and pCR.

**Supplementary Figure S2:** Correlation between pCR and DFS/EFS at landmark time points.

**Supplementary Table S1:** Population, intervention, comparison, outcomes, and study (PICOS)-based inclusion and exclusion criteria for studies included in the systematic review

| **Category** | **Inclusion criteria** | **Exclusion criteria** |
| --- | --- | --- |
| **Population** | Patients with HR+/HER2- breast cancer | - Non-human studies - Studies without outcomes for HR+/HER2- breast cancer patients - Studies including a mixed population were excluded if relevant data was not reported separately for the relevant patient population |
| **Intervention/**  **Comparators** | Any treatment in the adjuvant /neoadjuvant setting | Any study which did not have outcomes for adjuvant/neoadjuvant, setting |
| **Outcome** | pCR; EFS or DFS; OS | Studies not reporting any of the relevant outcomes |
| **Study design** | - RCTs - Non-RCTs, single arm trials - Prospective or retrospective observational studies - Studies with a sample size of > 30 | - Case studies, case reports, case series - Comments, editorials, narratives, letter to editor, opinion - Systematic literature reviews and meta-analyses were excluded but earmarked for bibliographic search |
| **Other criteria** | Studies in English, and published in or after 2000 | Non-English studies, and published prior to 2000 |

Abbreviations: DFS: disease-free survival; EFS: event-free survival; HR+/HER2-: hormone receptor positive and human epidermal growth factor-2 negative; OS: overall survival; pCR: pathological complete response; RCT: randomized controlled trials

**Supplementary Table S2:** Search strategy used for literature search

1. **Search strategy used for Medline (via Ovid) searched on January 5, 2021**

| **Concepts** | **#** | **Search Strategy** |
| --- | --- | --- |
| **Disease related terms** | 1 | exp breast neoplasms/ |
|  | 2 | (breast* or mammar*).ti,ab,kw. |
|  | 3 | (cancer* or neoplasm* or tumo?r* or oncolog* or malignan* or carcinoma* or sarcoma* or adenocarcinoma* or leiomyosarcoma*).ti,ab,kw. |
|  | 4 | 1 OR (2 AND 3) |
|  | 5 | (Estrogen receptor positive or er positive or er+ or er+ve or oestrogen receptor positive or progesterone receptor positive or pr positive or pr+ or pr+ve).ti,ab,kw. |
|  | 6 | (hr+ or hr positive or hr+ve or hormone receptor positive or hormone sensitive).ti,ab,kw. |
|  | 7 | 5 or 6 |
|  | 8 | (Erb-B2 Receptor Tyrosine Kinase 2 or Tyrosine Kinase-Type Cell Surface Receptor HER2 or Human Epidermal Growth Factor Receptor 2 or Receptor Tyrosine-Protein Kinase ErbB-2 or Proto-Oncogene C-ErbB-2 or Proto-Oncogene Neu).ti,ab,kw. |
|  | 9 | (human epidermal growth factor receptor type 2 negative or HER2-negative or HER2 negative or HER2- or HER2-ve).ti,ab,kw. |
|  | 10 | 8 or 9 |
|  | 11 | 7 AND 10 |
|  | 12 | 4 AND 11 |
| **Treatment setting related terms** | 13 | exp Chemotherapy, Adjuvant/ or exp Chemoradiotherapy, Adjuvant/ or exp Radiotherapy, Adjuvant/ or exp Neoadjuvant Therapy/ |
|  | 14 | ((adjuvant or neoadjuvant or neo-adjuvant or periadjuvant or peri-adjuvant) and (chemotherap* or therap* or treatment or setting?)).ti,ab,kw. |
|  | 15 | 13 or14 |
| **Outcomes related terms** | 16 | (pathological complete remission or pathological complete response or pathologic response or down-staging or pT0 or pCR or P0).ti,ab,kw. |
|  | 17 | exp disease free survival/ or (DFS or disease free survival or event free survival or efs).ti,ab,kw. |
|  | 18 | exp survival rate/ or (survival or overall survival or Survival Analysis or Survival Rate or OS).ti,ab,kw. |
|  | 19 | (16 and 17) or (16 and 18) or (17 and 18) |
| **Overall strategy** | 20 | 12 and 15 and 19 |
| **Limits** | 21 | limit 20 to yr="2000 - Current" |
|  | 22 | limit 21 to humans |
|  | 23 | limit 22 to english language |

1. **Search strategy used for Embase (via Ovid) searched on January 5, 2021**

| **Concepts** | **#** | **Search Strategy** |
| --- | --- | --- |
| **Disease related terms** | 1 | exp breast cancer/ |
|  | 2 | (breast* or mammar*).ti,ab,kw. |
|  | 3 | (cancer* or neoplasm* or tumo?r* or oncolog* or malignan* or carcinoma* or sarcoma* or adenocarcinoma* or leiomyosarcoma*).ti,ab,kw. |
|  | 4 | 1 OR (2 AND 3) |
|  | 5 | (Estrogen receptor positive or er positive or er+ or er+ve or oestrogen receptor positive or progesterone receptor positive or pr positive or pr+ or pr+ve).ti,ab,kw. |
|  | 6 | (hr+ or hr positive or hr+ve or hormone receptor positive or hormone sensitive).ti,ab,kw. |
|  | 7 | 5 or 6 |
|  | 8 | (Erb-B2 Receptor Tyrosine Kinase 2 or Tyrosine Kinase-Type Cell Surface Receptor HER2 or Human Epidermal Growth Factor Receptor 2 or Receptor Tyrosine-Protein Kinase ErbB-2 or Proto-Oncogene C-ErbB-2 or Proto-Oncogene Neu).ti,ab,kw. |
|  | 9 | (human epidermal growth factor receptor type 2 negative or HER2-negative or HER2 negative or HER2- or HER2-ve).ti,ab,kw. |
|  | 10 | 8 or 9 |
|  | 11 | 7 AND 10 |
|  | 12 | 4 AND 11 |
| **Treatment setting related terms** | 13 | exp cancer adjuvant therapy/ or exp adjuvant/ or exp adjuvant radiotherapy/ or exp adjuvant chemoradiotherapy/ or exp adjuvant therapy/ or exp adjuvant chemotherapy/ or exp neoadjuvant therapy/ or exp neoadjuvant chemotherapy/ |
|  | 14 | ((adjuvant or neoadjuvant or neo-adjuvant or periadjuvant or peri-adjuvant) and (chemotherap* or therap* or treatment or setting?)).ti,ab,kw. |
|  | 15 | 13 or14 |
| **Outcomes related terms** | 16 | (pathological complete remission or pathological complete response or pathologic response or down-staging or pT0 or pCR or P0).ti,ab,kw. |
|  | 17 | exp disease free survival/ or exp event free survival/ or (DFS or disease free survival or event free survival or efs).ti,ab,kw. |
|  | 18 | exp overall survival/ or (survival or overall survival or Survival Analysis or Survival Rate or OS).ti,ab,kw. |
|  | 19 | (16 and 17) or (16 and 18) or (17 and 18) |
| **Overall strategy** | 20 | 12 and 15 and 19 |
| **Limits** | 21 | limit 20 to yr="2000 - Current" |
|  | 22 | limit 21 to humans |
|  | 23 | limit 22 to english language |

1. **Search strategy used for Cochrane (via Cochrane Library) searched on January 5, 2021**

| **Concepts** | **#** | **Search Strategy** |
| --- | --- | --- |
| **Disease related terms** | 1 | [mh "breast neoplasms"] |
|  | 2 | (breast* OR mammar*):ti,ab,kw |
|  | 3 | (cancer* OR neoplasm* OR tumo?r* OR oncolog* OR malignan* OR carcinoma* OR sarcoma* OR adenocarcinoma* OR leiomyosarcoma*):ti,ab,kw |
|  | 4 | 1 OR (2 AND 3) |
|  | 5 | ("Estrogen receptor positive" OR "er positive" OR "er+" OR "er+ve" OR "oestrogen receptor positive" OR "progesterone receptor positive" OR "pr positive" OR "pr+" OR "pr+ve"):ti,ab,kw |
|  | 6 | ("hr+" OR "hr positive" OR "hr+ve" OR "hormone receptor positive" OR "hormone sensitive"):ti,ab,kw |
|  | 7 | 5 or 6 |
|  | 8 | ("Erb-B2 Receptor Tyrosine Kinase 2" OR "Tyrosine Kinase-Type Cell Surface Receptor HER2" OR "Human Epidermal Growth Factor Receptor 2" OR "Receptor Tyrosine-Protein Kinase ErbB-2" OR "Proto-Oncogene C-ErbB-2" OR "Proto-Oncogene Neu"):ti,ab,kw |
|  | 9 | ("human epidermal growth factor receptor type 2 negative" OR "HER2 negative" OR "HER2-" OR "HER2-ve"):ti,ab,kw |
|  | 10 | 8 or 9 |
|  | 11 | 7 AND 10 |
|  | 12 | 4 AND 11 |
| **Treatment setting related terms** | 13 | [mh "adjuvant chemotherapy"] OR [mh "adjuvant drug therapy"] OR [mh "neoadjuvant therapy"] OR [mh "neoadjuvant treatment"] OR [mh "neoadjuvant therapies"] OR [mh "neoadjuvant treatments"] |
|  | 14 | ((adjuvant OR neoadjuvant OR "neo adjuvant" OR periadjuvant OR "peri adjuvant") AND (chemotherap* OR therap* OR treatment OR setting?)):ti,ab,kw |
|  | 15 | 13 or14 |
| **Outcomes related terms** | 16 | ("pathological complete remission" OR "pathological complete response" OR "pathologic response" OR down-staging OR pT0 OR pCR OR P0):ti,ab,kw |
|  | 17 | [mh "Disease-Free Survival"] OR (DFS OR "disease-free survival" OR "disease free survival" OR "event-free survival" OR "event free survival" OR efs):ti,ab,kw |
|  | 18 | [mh "Survival Rate"] OR (survival OR "overall survival" OR "survival rate" or "survival analysis" OR OS):ti,ab,kw |
|  | 19 | (16 and 17) or (16 and 18) or (17 and 18) |
| **Overall strategy** | 20 | 12 and 15 and 19 |
| **Limits** | 21 | #21 with Cochrane Library publication date between Jan 2000 and Jan 2021 |
|  | 22 | [mh humans] |
|  | 23 | ("Human" OR "Humans" OR "Homo sapiens" OR "Homo-sapiens" OR "Person" OR "Persons" OR "Human race" OR "Human-race" OR "Human being" OR "Human being" OR "Human-being" OR "Human-beings") |
|  | 24 | #22 OR #23 |
|  | 25 | #21 AND #24 |

**Supplementary Table S3:** Definitions of DFS, EFS, RFS, etc. used across various studies

| **Study** | **End point** | **Definition** |
| --- | --- | --- |
| Thangarajah, 2017 | DFS | Time from diagnosis to local recurrence, recurrence in axillary lymph nodes, distant metastasis or death |
| Nonneville, 2019 | DFS | Time from surgery to relapse (breast, node or distant) or death from any cause |
| Mackey, 2013 | DFS | Time from randomization to the date of a clinical relapse |
| Park, 2017 | DFS | NA |
| Lobefaro 2020 | DFS | Time from surgery to locoregional recurrence or distant recurrence or patient death from any cause |
| De Nonneville 2018 | DFS | NA |
| Babacan, 2015 | DFS | NA |
| Conte, 2020 | DFS | Time from the date of randomization to the date of local recurrence or distant metastases or contralateral/ipsilateral breast tumor |
| Zhu 2019 | DFS | Time from surgery to local recurrence or distant metastasis |
| Fujii 2016 | RFS | NA |
| Fehrenbacher 2020 | IDFS | Time from random assignment to local invasive recurrence after mastectomy, local invasive recurrence in the ipsilateral breast after lumpectomy, regional recurrence, distant recurrence, contralateral invasive breast cancer, second non-breast primary cancer (excluding squamous or basal cell carcinoma of the skin), or death from any cause before recurrence or second primary cancer |
| Liu 2017 | EFS | Months from date of diagnosis to date of first event or death or last follow-up in those without events |
| O'Shaughnessy 2015 | DFS | Time from randomization until recurrence or death |
| Ruiz-Borrrego, 2019 | DFS | Time from the date of randomization to the date of local or distant invasive recurrence of breast cancer or second primary breast cancer or death from any cause |
| Koh 2014 | RFS | Time from surgery to disease relapse and death from related cause; the follow-up of patients still alive has been censored at their latest date of follow-up. |
| Pippen 2011 | DFS | NA |
| Cheang 2012 | RFS | Time from trial arm random assignment to any recurrences including local breast chest wall, regional, or distant relapses |
| Schneeweiss A. 2020 | IDFS | Defined as the time period between registration and first event and will be analyzed after the end of the study by referring to data from GBG patient's registry |
| Balduzzi 2014 | DFS | Time from surgery to any subsequent relapse (including ipsilateral breast recurrence, contralateral breast cancer, and appearance of a second primary cancer) or death |
| Sparano 2018 | DFS | Disease-free survival (DFS) is defined to be time from randomization to first event, where the first event is any of ipsilateral breast tumor recurrence, local recurrence, regional recurrence, distant recurrence, contralateral second primary invasive cancer, second primary non-breast invasive cancer (excluding non-melanoma skin cancers), or death without evidence of recurrence |
| Sparano 2015 | DFS | Defined as local, regional, and/or distant relapse, second primary breast cancer, or death without recurrence. |
| Saigosoom 2020 | DFS | NA |
| Choi 2010 | DFS | Time from the date of diagnosis to the date of the documentation of relapse, including locoregional recurrence and/or distant metastasis |
| Ebner 2015 | DFS | NA |
| Fountzilas 2012 | DFS | DFS was measured from the date of randomization until recurrence of tumor or secondary neoplasm or death from any cause |
| Sinn, 2019 | DFS | Disease-free survival (DFS) was defined as time from study entry to local or distant recurrence or death from any cause, distant recurrence free survival |
| Minckwitz 2013 | DFS | Defined as the interval between start of TAC chemotherapy and occurrence of a first event (All invasive relapses and all deaths) |
| von Minckwitz 2014 | DFS | DFS was defined as time from randomization or histological diagnosis in those patients not being randomized for the bevacizumab question until any invasive locoregional, invasive contralateral or distant recurrence of breast cancer or any second primary invasive non-breast cancer or death due to any cause. |
| Nanda, 2020 | EFS | NA |
| Kim 2010 | DFS | DFS was calculated as the time from the date of surgery to the date of the development of local, regional and distant metastases, and the date of death before recurrence. |
| Gwark 2020 | RFS | RFS was defined as the time from the date of the study enrollment to the first date of disease recurrence. |
| Zhang, 2013 | DFS | Time interval between the date of definitive surgery and the date of a first recurrence event (locoregional or |
| Groheux, 2015 | EFS | Time from PET scan to local, regional, or distant recurrences or death, whichever occurred first. |
| Darb-Esfahani, 2009 | DFS | NA |
| Kogawa, 2018 | RFS | Locoregional recurrence, distant metastases, and death without disease recurrence were considered to be a recurrence-free survival (RFS) event. Contralateral breast cancers were not considered to be recurrence events in this study. RFS time was defined as the period between the date of definitive surgery and the date of disease recurrence or death or last follow-up. |
| Viala, 2018 | PFS | Survival was defined from the date of diagnosis to the date of relapse (PFS) or date of death |
| Masuda 2013 | DFS | DFS time from the date of definitive surgery to the date of recurrence from the original cancer. |
| Vargo 2011 | DFS | Disease-free survival (DFS) was calculated from the date of surgery to the date of first recurrence (locoregional or distant). |
| Esserman 2012 | RFS | RFS was calculated from the date of chemotherapy initiation. |
| Bonnefoi 2014 | EFS | Progression or relapse while on neoadjuvant chemotherapy, Site of first loco-regional recurrence, Site of first distant (Soft tissue, Visceral, Skeletal, CNS and others) recurrence, and death without prior report of progression were considered as event for EFS. |
| Guiu 2013 | DFS | Disease-free survival (DFS) was defined as the date of the first histology to the date of the first recurrence of breast cancer at any site, or death from any cause, or the date of a second cancer. |
| Krishnan 2013 | DFS | DFS was measured from the date of first diagnosis to the date of first local or distant metastasis or last follow-up. |
| Ohzawa 2014 | DFS | NA |
| Fukada 2018 | DFS | NA |
| Demir, 2013 | RFS | Recurrence-free survival (RFS) was defined as the time (months) from the diagnosis of locally advanced breast cancer to time to progression or distant metastasis or the final visit (whichever came first) |
| Kim 2016 | RFS | Calculated from the start of Neoadjuvant chemotherapy to recurrence of breast cancer |
| Bae 2016 | DFS | DFS was measured as the period between breast cancer diagnosis and first tumor recurrence, including locoregional recurrence, and distant recurrence. |
| Cabrera-Galeana 2020 | DFS | DFS was calculated from the time of diagnosis to the date of first documentation of recurrent disease or last relapse‐free visit |
| Villarreal-Garza 2016 | DFS | DFS was calculated from the time of surgery to the date of first identification of recurrent disease or last relapse-free visit, and |
| Hayashi 2020 | DFS | Disease-free survival (DFS) was calculated from the date of surgery to local or distant recurrence, death, or last follow-up |
| Cho 2019 | DFS | DFS was defined as the time from the initiation of NAC to any recurrence or death. |
| Loo 2016 | RFS | Invasive ipsilateral breast tumor recurrence, local/regional Invasive Recurrence, distant recurrence, death from breast cancer, death from non-breast cancer cause, death from unknown cause |
| Marme 2016 | DFS | calculated from the date of registration to the date of locoregional, distant relapse, or death of any cause (DFS) |
| Wang 2016 | DFS | Disease-free survival (DFS) refers to the time from start of neoadjuvant chemotherapy to the appearance of local recurrence, regional metastasis, second primary cancer, distant metastasis, or death. |
| Choi 2018 | DFS | NA |
| Bonnefoi, 2019 | RFI | Time from randomization to progression on chemotherapy, ipsilateral invasive breast (local) recurrence, regional recurrence, distant recurrence or death due to breast cancer and/or treatment toxicity, whichever came first. |
| Collins 2020 | iDF | defined as freedom from invasive disease recurrence, second primary cancer, or death. |
| Battisti, 2020 | DFS | DFS was calculated as the time from surgery until disease recurrence. Disease-free patients were censored at the last follow-up date. |
| Borges | DFS | NA |
| Jevric 2019 | DFS | Time from radical breast surgery to loco-regional recurrence and/or distant metastases and/or contralateral breast cancer |
| Ignatiadis 2007 | DFS | The time from study entry until the day of the first evidence of disease recurrence either locoregional or distant (disease-free survival [DFS]) |
| Fountzilas, 2012 | DFS | DFS was measured from the date of randomization until recurrence of tumor or secondary neoplasm or death from any cause |
| Xue, 2012 | DFS | DFS was defined as the interval from the first treatment for breast cancer to the first recurrence (locoregional relapse, distant metastasis, or contralateral breast). |
| Batra, 2020 | iDFS | iDFS as time from diagnosis of breast cancer to local, regional or distant recurrence, invasive contralateral breast cancer, second primary cancer (except non-melanoma skin cancer and in-situ cancer), or death due to any cause. |
| Baulies, 2015 | DFS | NA |
| Yamada, 2018 | RFS | RFS was defined as the time from the operation day to the first of one of the following events: recurrence at local, regional, or distant sites; or a new invasive cancer in the contralateral breast. |
| Cancello 2010 | DFS | DFS was defined as the length of time from the date of surgery to any relapse (including ipsilateral breast recurrence), the appearance of a second primary cancer (including contralateral breast cancer) or death, whichever occurred first. |
| Loi 2013 | DFS | Disease-free survival (DFS) was defined as time from date of random assignment to date of first relapse (local, regional, contralateral, or metastatic), second primary malignancy, or death resulting from any cause (whichever occurred first). |
| Munzone 2014 | DFS | DFS was defined as the time from surgery to events such as relapse (including ipsilateral breast recurrence), appearance of a second primary cancer (including contralateral breast cancer), or death, whichever occurred first |
| Valentina 2017 | DFS | DFS was defined as the time from diagnosis of BC to time from surgery to any invasive or non-invasive BC recurrence, either local, regional, contralateral or distant. |
| Clark 2018 | DFS | NA |
| Sparano 2012 | DFS | Disease-free survival was defined as the time from randomization to disease recurrence, diagnosis of contralateral breast cancer, or death from any cause, whichever occurred first. |
| Loibl 2018 | OS | overall survival (OS) defined as the time from randomization to death due to any cause. |
| Liu 2008 | DFS | Disease-free survival was defined as the time between the date |
| Skarlos 2012 | DFS | DFS was measured from the date of randomization until recurrence of the tumor, or secondary neoplasm, or death from any cause. |
| Grassadonia, 2014 | DFS | DFS as the time between surgery and the first verified event. |
| Elzawahry 2013 | DFS | DFS was defined as the time between the date of curative surgery and date of first relapse (local or distant) or death |
| Kolberg 2017 | DFS | DFS was defined as any relapse or death. Patients were censored at the time of last follow-up |
| Papaxoinis 2015 | DFS | Disease-free survival (DFS) was measured from the date of diagnosis until verified disease progression, death or last contact, whichever occurred first |
| Cardoso 2016 | DFS | Disease-free survival was defined as the time until first disease progression (locoregional, distant relapse, ipsilateral or contralateral invasive breast cancer, ductal carcinoma in situ, or an invasive second primary cancer) or death from any cause |
| Vaz-Luis 2014 | iDFS | Invasive ipsilateral tumor recurrence, Locoregional invasive recurrence (Chest wall), Distant recurrence (Liver, Lung, Other distant visceral, Pleural effusion, Distant lymph nodes, Bone and Skin), Other causes of death and Breast cancer-specific death were considered as IDFS event. |
| Waintraub 2019 | RFS | NA |
| Zeng 2018 | DFS | NA |
| Fountzilas 2015 | DFS | NA |
| Goldvaser 2017 | DFS | Disease free survival (DFS) was defined as the time between surgery to event (recurrence or death) or end of follow-up. |
| Cardoso 2017 | DFS | Disease-free survival was defined as the time until first disease progression (locoregional, distant relapse, ipsilateral or contralateral invasive breast cancer, ductal carcinoma in situ, or an invasive second primary cancer) or death from any cause |
| Cardoso 2018 | DFS | Disease-free survival was defined as the time until first disease progression (locoregional, distant relapse, ipsilateral or contralateral invasive breast cancer, ductal carcinoma in situ, or an invasive second primary cancer) or death from any cause |
| Cardoso 2019 | DFS | Disease-free survival was defined as the time until first disease progression (locoregional, distant relapse, ipsilateral or contralateral invasive breast cancer, ductal carcinoma in situ, or an invasive second primary cancer) or death from any cause |
| Nadia Harbeck 2020 | iDFS | NA |

**Supplementary Table S4.** Correlation between OS at yearly landmark time points and pCR

| Model | Number of observations | Number of patients | R^2^  (95% CI) | r  (95% CI) | *p*-value |
| --- | --- | --- | --- | --- | --- |
| 1-yr OS vs. pCR | 31 | 19,385 | 0.02  (0, 0.2) | 0.15  (-0.22, 0.48) | 0.42 |
| 2-yr OS vs. pCR | 31 | 19,385 | 0.02  (0, 0.2) | 0.15  (-0.22, 0.48) | 0.42 |
| 3-yr OS vs. pCR | 34 | 19,628 | 0.02  (0, 0.18) | 0.14  (-0.21, 0.46) | 0.43 |
| 4-yr OS vs. pCR | 33 | 19,545 | 0.03  (0, 0.22) | 0.18  (-0.17, 0.5) | 0.30 |
| 5-yr OS vs. pCR | 33 | 23,192 | 0.11  (0, 0.32) | 0.33  (-0.01, 0.61) | 0.06 |

Abbreviations: CI, confidence interval; OS, overall survival; pCR, pathological complete response; R^2^, coefficient of determination; r, Pearson’s correlation coefficient; yr, year

| Model | Number of observations | N | R^2^  (95% CI) | r  (95% CI) | *p*-value |
| --- | --- | --- | --- | --- | --- |
| 1-yr DFS/EFS vs. pCR | 35 | 11,691 | 0  (0, 0.06) | 0.02  (-0.32, 0.35) | 0.93 |
| 2-yr DFS/EFS vs. pCR | 36 | 11,950 | 0.01  (0, 0.14) | -0.09  (-0.4, 0.25) | 0.61 |
| 3-yr DFS/EFS vs. pCR | 35 | 11,691 | 0.01  (0, 0.14) | -0.08  (-0.4, 0.26) | 0.66 |
| 4-yr DFS/EFS vs. pCR | 36 | 11,813 | 0  (0, 0.09) | 0.03  (-0.3, 0.36) | 0.84 |
| 5-yr DFS/EFS vs. pCR | 32 | 11,134 | 0  (0, 0.14) | -0.07  (-0.4, 0.29) | 0.72 |

**Supplementary Table S5.** Correlation between DFS/EFS at yearly landmark time points and pCR

Abbreviations: CI, confidence interval; DFS, disease-free survival; EFS, event-free survival; OS, overall survival; pCR, pathological complete response; R^2^, coefficient of determination; r, Pearson’s correlation coefficient; yr, year

**Supplementary Table S6.** Sensitivity analysis of correlation between OS and DFS/EFS at landmark time points

| Model | Scenario | R^2^  (95% CI) | r  (95% CI) | *p*-value |
| --- | --- | --- | --- | --- |
| 3-yr OS vs. 1-yr DFS/EFS | Full dataset | 0.48  (0.33, 0.59) | 0.7  (0.57, 0.79) | <.0001 |
|  | After removing outliers | 0.39  (0.23, 0.52) | 0.63  (0.48, 0.74) | <.0001 |
| 4-yr OS vs. 1-yr DFS/EFS | Full dataset | 0.5  (0.35, 0.61) | 0.71  (0.59, 0.8) | <.0001 |
|  | After removing outliers | 0.43  (0.26, 0.55) | 0.65  (0.51, 0.76) | <.0001 |
| 4-yr OS vs. 2-yr DFS/EFS | Full dataset | 0.57  (0.43, 0.66) | 0.76  (0.65, 0.83) | <.0001 |
|  | After removing outliers | 0.51  (0.36, 0.62) | 0.72  (0.59, 0.81) | <.0001 |
| 5-yr OS vs. 1-yr DFS/EFS | Full dataset | 0.48  (0.32, 0.59) | 0.69  (0.56, 0.79) | <.0001 |
|  | After removing outliers | 0.36  (0.2, 0.49) | 0.6  (0.45, 0.72) | <.0001 |
| 5-yr OS vs. 2-yr DFS/EFS | Full dataset | 0.56  (0.42, 0.65) | 0.75  (0.64, 0.82) | <.0001 |
|  | After removing outliers | 0.49  (0.33, 0.6) | 0.7  (0.57, 0.79) | <.0001 |
| 5-yr OS vs. 3-yr DFS/EFS | Full dataset | 0.58  (0.44, 0.67) | 0.76  (0.66, 0.84) | <.0001 |
|  | After removing outliers | 0.46  (0.3, 0.57) | 0.68  (0.54, 0.78) | <.0001 |

Abbreviations: CI, confidence interval; DFS, disease-free survival; EFS, event-free survival; OS, overall survival; pCR, pathological complete response; R^2^, coefficient of determination; r, Pearson’s correlation coefficient; yr, year

**Supplementary Table S7.** Sensitivity analysis of correlation between pCR and DFS at landmark time points

| Model | Scenario | R^2^  (95% CI) | r  (95% CI) | *p*-value |
| --- | --- | --- | --- | --- |
| pCR vs. 3-yr DFS | Full dataset | 0.01  (0, 0.14) | -0.08  (-0.4, 0.26) | 0.6575 |
|  | pCR = ypT0/is ypN0 | 0  (0, 0.1) | 0.03  (-0.36, 0.41) | 0.8908 |
|  | After removing outliers | 0  (0, 0.06) | -0.02  (-0.37, 0.35) | 0.9309 |
| pCR vs. 4-yr DFS | Full dataset | 0  (0, 0.09) | 0.03  (-0.3, 0.36) | 0.8413 |
|  | pCR = ypT0/is ypN0 | 0.01  (0, 0.16) | 0.08  (-0.31, 0.44) | 0.7095 |
|  | After removing outliers | 0  (0, 0.1) | 0.03  (-0.32, 0.38) | 0.8524 |
| pCR vs. 5-yr DFS | Full dataset | 0  (0, 0.14) | -0.07  (-0.4, 0.29) | 0.7217 |
|  | pCR = ypT0/is ypN0 | 0.02  (0, 0.22) | 0.13  (-0.29, 0.51) | 0.5345 |
|  | After removing outliers | 0.01  (0, 0.17) | 0.09  (-0.28, 0.45) | 0.6278 |

Abbreviations: CI, confidence interval; DFS, disease-free survival; pCR, pathological complete response; R^2^, coefficient of determination; r, Pearson’s correlation coefficient; yr, year

**Supplementary figure legends**


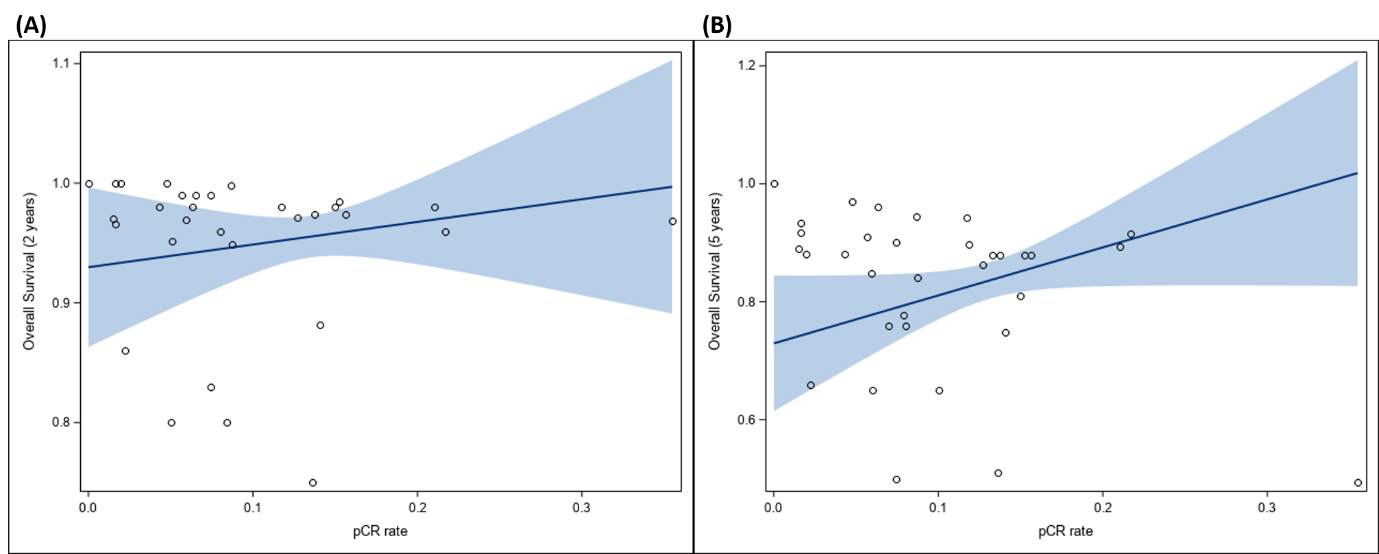


**Supplementary Figure S1.** Correlation between rates of OS at landmark time points and pCR. (A) 2-yr rates of OS vs pCR. (B) 5-yr rates of OS vs pCR

pCR, pathological complete response; OS, overall survival; yr, year


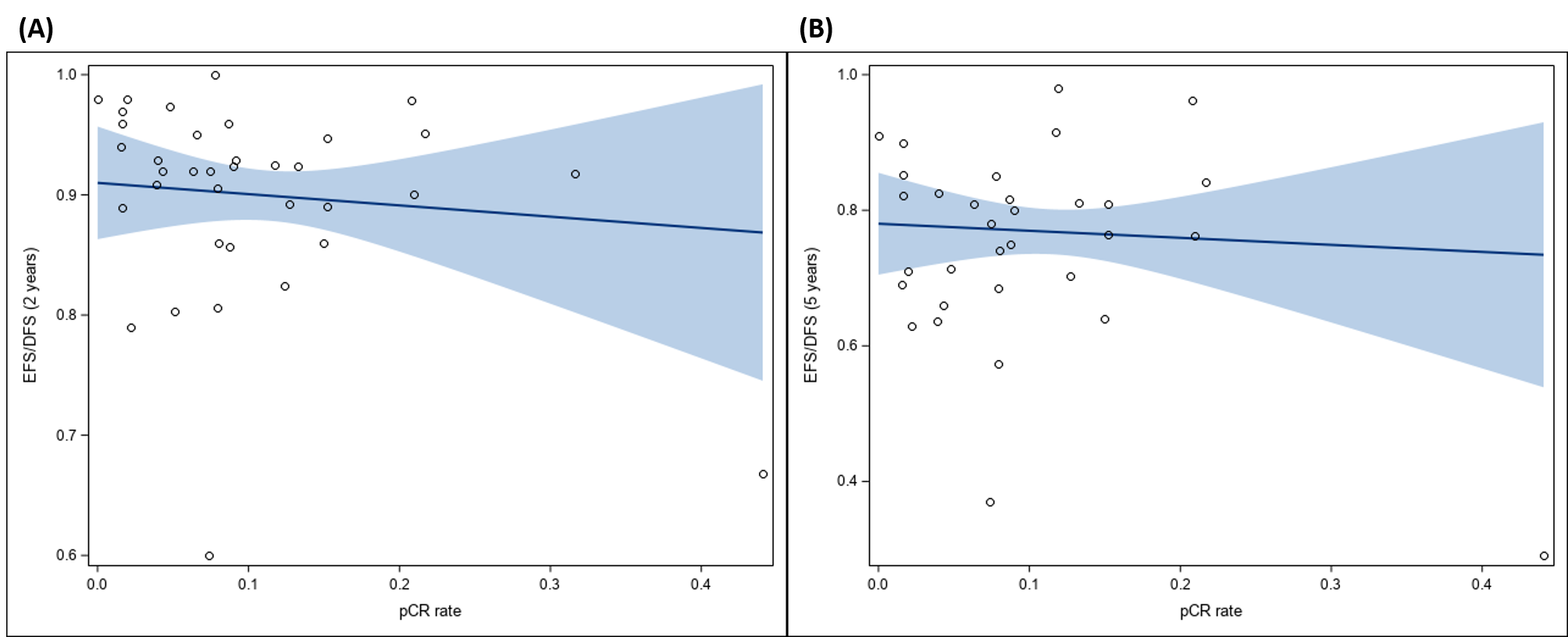


**Supplementary Figure S2.** Correlation between pCR and DFS/EFS at landmark time points. (A) 2-yr rates of DFS/EFS vs pCR. (B) 5-yr rates of DFS/EFS vs pCR

DFS, disease-free survival; EFS, event-free survival; pCR, pathological complete response; yr, year
